# The short-term effects of planktivorous fish foraging in the presence of artificial light at night on lake zooplankton

**DOI:** 10.1093/plankt/fbac046

**Published:** 2022-09-07

**Authors:** Joanna Tałanda, Piotr Maszczyk, Ewa Babkiewicz, Katarzyna Rutkowska, Mirosław Ślusarczyk

**Affiliations:** Department of Hydrobiology, Institute of Functional Biology and Ecology, Faculty of Biology, University of Warsaw at Biological and Chemical Research Centre, żwirki i Wigury 101, 02-089 Warsaw, Poland; Department of Hydrobiology, Institute of Functional Biology and Ecology, Faculty of Biology, University of Warsaw at Biological and Chemical Research Centre, żwirki i Wigury 101, 02-089 Warsaw, Poland; Department of Hydrobiology, Institute of Functional Biology and Ecology, Faculty of Biology, University of Warsaw at Biological and Chemical Research Centre, żwirki i Wigury 101, 02-089 Warsaw, Poland; Department of Hydrobiology, Institute of Functional Biology and Ecology, Faculty of Biology, University of Warsaw at Biological and Chemical Research Centre, żwirki i Wigury 101, 02-089 Warsaw, Poland; Department of Hydrobiology, Institute of Functional Biology and Ecology, Faculty of Biology, University of Warsaw at Biological and Chemical Research Centre, żwirki i Wigury 101, 02-089 Warsaw, Poland

**Keywords:** ALAN, community structure, light pollution, size-selective predation, size structure, zooplankton

## Abstract

Numerous studies have revealed that artificial light at night alters the natural patterns of light in space and time and may have various ecological impacts at different ecological levels. However, only a few studies have assessed its effect on interactions between organisms in aquatic environments, including predator–prey interactions in lakes. To fill this gap, we performed a preliminary enclosure experiment in which we compared the foraging effect of juvenile perch (*Perca fluviatilis*) on a natural lake zooplankton community in the absence and presence of light of high-pressure sodium (HPS) lamps mimicking artificial light emitted by a boat. The results revealed that even short-lasting exposure to HPS lamps may result in increasing fish predation, which in turn decreased the mean body size in zooplankton populations (e.g. *Bosmina thersites*) and affected the relative proportion between different taxa in zooplankton communities.

## INTRODUCTION

Artificial light at night (ALAN) alters the natural patterns of light in space and time, disrupting natural resource use and information flow. This may have ecological impacts at the individual, population and community levels (e.g. Longcore and Rich, 2004; Sanders *et al.,* 2021; Zapata *et al.,* 2019). Studies on the effects of ALAN at the population and community levels have been focused on interactions between organisms, including relations between a predator and its prey (e.g. Czarnecka *et al.,* 2019; Fermin and Seronay, 1997; Nguyen and Winger, 2019; Nuñez *et al.,* 2021). However, these effects in relation to the effects at the individual level remain relatively understudied (Gaston *et al.,* 2015; Nuñez *et al.,* 2021; Owens and Lewis, 2018; Sanders and Gaston, 2018). This issue is poorly explored even for planktivorous fish and zooplankton interactions—the best studied model of predator–prey interplay.

In lakes inhabited by fish, the foraging activity of visually oriented positive-size selective fishes is the main driver shaping space distribution, population size, age structure and community composition of planktonic animals (e.g. Gliwicz, 2003). ALAN by disrupting natural light intensity and spectral composition could alter the impact of fish predation on zooplankton and in turn on their population and community structure. However, the literature provides mainly indirect evidence supporting this prediction. ALAN was shown to attract fish (e.g. Becker *et al.,* 2013; Nguyen and Winger, 2019) and enhance their foraging efficiency (Bolton *et al.,* 2017; Fermin and Seronay, 1997). From the zooplankton perspective, it has been revealed that its response to the mortality risk in the presence of ALAN may be maladaptive (Maszczyk *et al.,* 2021; Tałanda *et al.,* 2018). Direct evidence concerns only the ALAN-dependent effects of fish predation on depth selection behavior of zooplankton populations and communities (Fermin and Seronay, 1997; Maszczyk *et al.,* 2021; Moore *et al.,* 2000; Springer and Skrzypczak, 2015), but neither on the mean body size nor community composition. The aim of our study was to fill this gap by assessing the short-term ALAN-dependent effect of a foraging planktivorous fish lake zooplankton body size and community composition in a preliminary enclosure experiment. More specifically, we hypothesized that the presence of artificial light produced by the high-pressure sodium (HPS) lamp may affect zooplankton vertical distribution and may increase positive size selective fish predation, which in turn decreases both the mean body size in zooplankton populations and the relative density of more vulnerable (i.e. larger-bodied and less evasive) species in zooplankton communities.

## METHODS

**Fig. 1:**
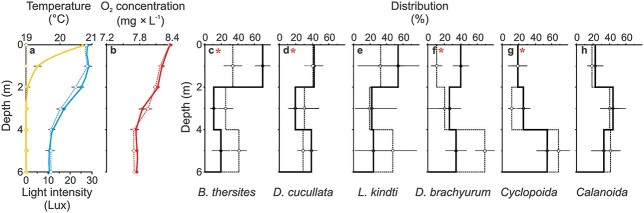
The gradients of light intensity (yellow lines and circles, a), temperature (blue lines and circles, a), oxygen concentration (red lines and circles, b) and density of different taxonomic groups of planktonic animals (black lines and circles, c–h) as the mean (±1SD) for all enclosures in the “dark” (dotted lines and open circles) and in “ALAN” treatment (continuous lines and filled circles). Significant differences for comparisons between the two treatments (“dark” and “ALAN”) across all depths are shown as *, according to 95% CI and the bootstrap method.

The experiment was conducted on July 22–23, 2017 during the new moon in a small and shallow (7.0 m) bay located far from ALAN sources in the thermally stratified (Fig. 1), eutrophic (chlorophyll *a* conc. = 8.31 *μ*g L^−1^, Secchi disc = 2.6 m) Lake Roś (53°40′24.4”N 21°53′52.7″E; Great Mazurian Lakes; NE Poland). At the time of the experiment, the zooplankton community was dominated in decreasing order by *Cyclopoida*, *Calanoida*, *Daphnia cucullata* and *Bosmina thersites*. The remaining taxa were less represented by at least an order of magnitude. The enclosures were made of transparent PVC (H = 7.7 m, ⌀ = 0.5 m), open at the top and closed at the bottom, and were attached to an anchored floating platform with an HPS artificial light source (70 W OSRAM®). The light source was attached to a non-transparent lampshade set at 2.4 m above water, mimicking the light emitted by a boat on a lake and producing approximately 8× greater light intensity and an entirely different spectral composition (e.g. Maszczyk *et al.,* 2021) than full moonlight (Appendix 1). Three enclosures were placed on the “bright” side of the platform, while the 3 others were covered at the top with black non-transparent tarpaulin and placed on the opposite “dark” side. We used the zooplankton community originating as prey (collected from the whole water column in the experimental bay with a plankton net of 150-μm mesh size), and juvenile 0+ individuals of visually oriented planktivore fish *Perca fluviatilis* as a predator, all originating from Lake Roś. The fish were kept without food for 48 h before the experiment.

The day before the experiment, the enclosures were filled with filtered (5-μm mesh size) lake water (pumped by a petrol-driven water pump from a 1-m depth) and left for 5 hours to establish a natural temperature gradient. The zooplankton was introduced from the lake into the enclosures in natural densities at 7 p.m. At 12 p.m., vertical gradients of physical and chemical parameters of the water were measured in the enclosures (Fig. 1), and non-stratified samples of zooplankton from each of the enclosures were collected (integrated vertical sample). After that, the “dark” enclosures were covered with a tarpaulin and the light was switched on and then measured in each enclosure (Fig. 1). The experiment started at 1 a.m. (three hours after sunset) by adding 20 perch to each of the enclosures. At 4 a.m. (dawn), zooplankton stratified samples (0–2, 2–4 and 4–6 m) were collected from each enclosure, the light was switched off and then all fish were caught, euthanized, preserved in ethanol and transported to the lab, where their gastrointestinal tracts were dissected.

Zooplankton samples (initial and final) and fish gastrointestinal tracts from each of the enclosures (*n* = 16.3 ± 2.1 and 17.3 ± 1.5 in dark and ALAN treatments) were analyzed under a stereomicroscope by counting and assigning individuals to six of the most numerous taxa (cladocerans: *B. thersites, D. cucullata*, *Leptodora kindtii*, *Diaphanosoma brachyurum* and copepods: *Cyclopoida sp.*, *Calanoida sp.*). The length of 30 randomly selected individuals of *B. thersites* individuals (the most common taxa in the fish intestines) was also measured in each zooplankton sample. Statistical comparisons of the measured parameters between the two tested treatments were performed with a bootstrap approach that was used to simulate the distributions of the analyzed variables with the use of the collected data. The 95% bias-corrected confidence intervals were calculated for the differences between the illuminated and dark enclosures. Analyses of some parameters (e.g. taxon densities, body size of *B. thersites*) were performed on relative values considering the initial and final data in each enclosure. More details concerning the statistical procedures can be found in the Appendix 2.

## RESULTS AND DISCUSSION

The data for all of the six taxa assessed together revealed that zooplankton resided closer to the water surface in illuminated enclosures compared with the dark ones (Fig. 1, Table 1 in the Appendix 1), which is in the line with several previous studies (e.g. Fermin and Seronay, 1997; Springer and Skrzypczak, 2015; Szczerbowski and Mamcarz, 1984), but opposite to some others, which revealed a negative effect of ALAN on the depth distribution of zooplankton (Ludvigsen *et al.,* 2018; Maszczyk *et al.,* 2021; Moore *et al.,* 2000; Rudstam *et al.,* 1992). The observed variability could be attributed to differences in the light beam intensity, focus and variability (directed towards the water surface in the former studies or diffused and temporary variable in the latter ones). The positive phototaxis in our study may most likely be due to the maladaptive response of *Daphnia* perceiving the higher concentration of artificial light as a hint of the location of higher food concentration or temperature. More detailed analysis of our results revealed positive phototaxis to ALAN only in the case of smaller-bodied zooplankton taxa such as *B. thersites*, *D. brachyurum* and more evasive *Cyclopoida*, which are less vulnerable to positive size selective fish predation, but revealed negative phototaxis in the case of *D. longispina* and no effect in the case of remaining taxa (*Leptodora kindti* and *Calanoida*). This may suggest that the depth selection behavior of zooplankton depends also on the ALAN-dependent perceived mortality risk from predation. It should be pointed out that even when some taxa exhibit negative phototaxis to ALAN, their response may be too weak in relation to the real light-dependent mortality risk and in turn be maladaptive (Maszczyk *et al.,* 2021).

**Fig. 2:**
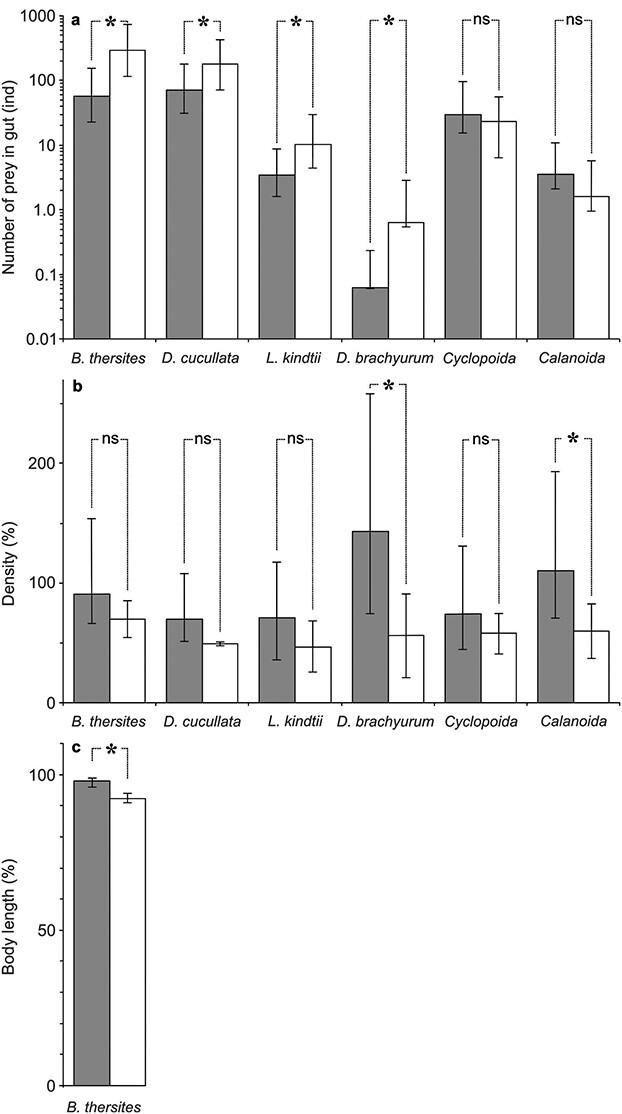
Digestive tract content of *P. fluviatilis* (a), % density of zooplankton after the experiment relative to that before the experiment across all depths (b) and % body length of *B. thersites* after the experiment relative to that before the experiment across all depths (c), in “dark” and “ALAN” enclosures (mean for all enclosures ±1SD). Significant differences for comparisons are shown as *, according to 95% CI and the bootstrap method. NS stands for non-significant.

The number of each of the four taxa of cladocerans, their sum and the sum of all of the six analyzed taxa was greater in the guts of fish inhabiting illuminated enclosures compared with the dark ones (Fig. 2a, Table 2 in the Appendix 1), which may be attributed to both attraction of zooplankton to the ALAN and their greater visibility in the presence of ALAN and in turn enhanced predation by visually oriented fish (Fermin and Seronay, 1997). Contrarily, the effect of ALAN on the number of eaten *Cyclopoida* and *Calanoida* was negligible, which may be due to their greater evasiveness in relation to the remaining zooplankton taxa.

While the final population density appears lower in all six analyzed taxa in the ALAN treatment than in the dark (Fig. 2b), a significant difference was evidenced in the case of *Calanoida* and *D. brachyurum* merely (Table 3 in the Appendix 1), most likely due to the small number of replicates in the experiment. Therefore, the results confirm our predictions that the presence of ALAN may affect the zooplankton community composition but did not confirm that its presence may result in a relative decrease in the density of more vulnerable, i.e. larger-bodied and less evasive species (such as *D. longispina* and *L. kindtii*).

The mean body length of the *B. thersites* population decreased during the experiment in both treatments, yet the difference was more apparent in the illuminated enclosures than in the dark ones (bootstrap distribution, mean bootstrap diff. = −0.05, low CI limit = −0.07, high CI limit = − 0.03, Fig. 2c), which may suggest greater positive-size selectivity in the artificially illuminated areas.

## CONCLUSIONS

Our results revealed that even short-lasting exposure to ALAN may affect the vertical distribution of lake zooplankton and increase fish foraging efficiency, which may affect the size structure of zooplankton populations and the structure of zooplankton communities, thus possibly affecting the functioning of the ecosystem as a whole.

## AUTHORS’ CONTRIBUTIONS

Conceptualization: J.T., M.Ś. and P.M., methodology: J.T., M.Ś.; validation: J.T., M.Ś. and P.M.; formal analysis, J.T., K.R.; investigation, J.T., M.Ś., P.M. and E.B.; writing: J.T., M.Ś. and P.M., funding acquisition, J.T. and P.M.

## Supplementary Material

Appendix_1_fbac046

Appendix_2_fbac046
