# Supplementary material for: The short-term effects of planktivorous fish foraging in the presence of artificial light at night on lake zooplankton
Source: J Plankton Res. 2022 Sep 7;44(6):942–6. doi: 10.1093/plankt/fbac046 (PMC9692195; doi:10.1093/plankt/fbac046)
Supplement: Appendix_1_fbac046 [file appendix_1_fbac046.docx]

**Appendix 1. The experimental setup**


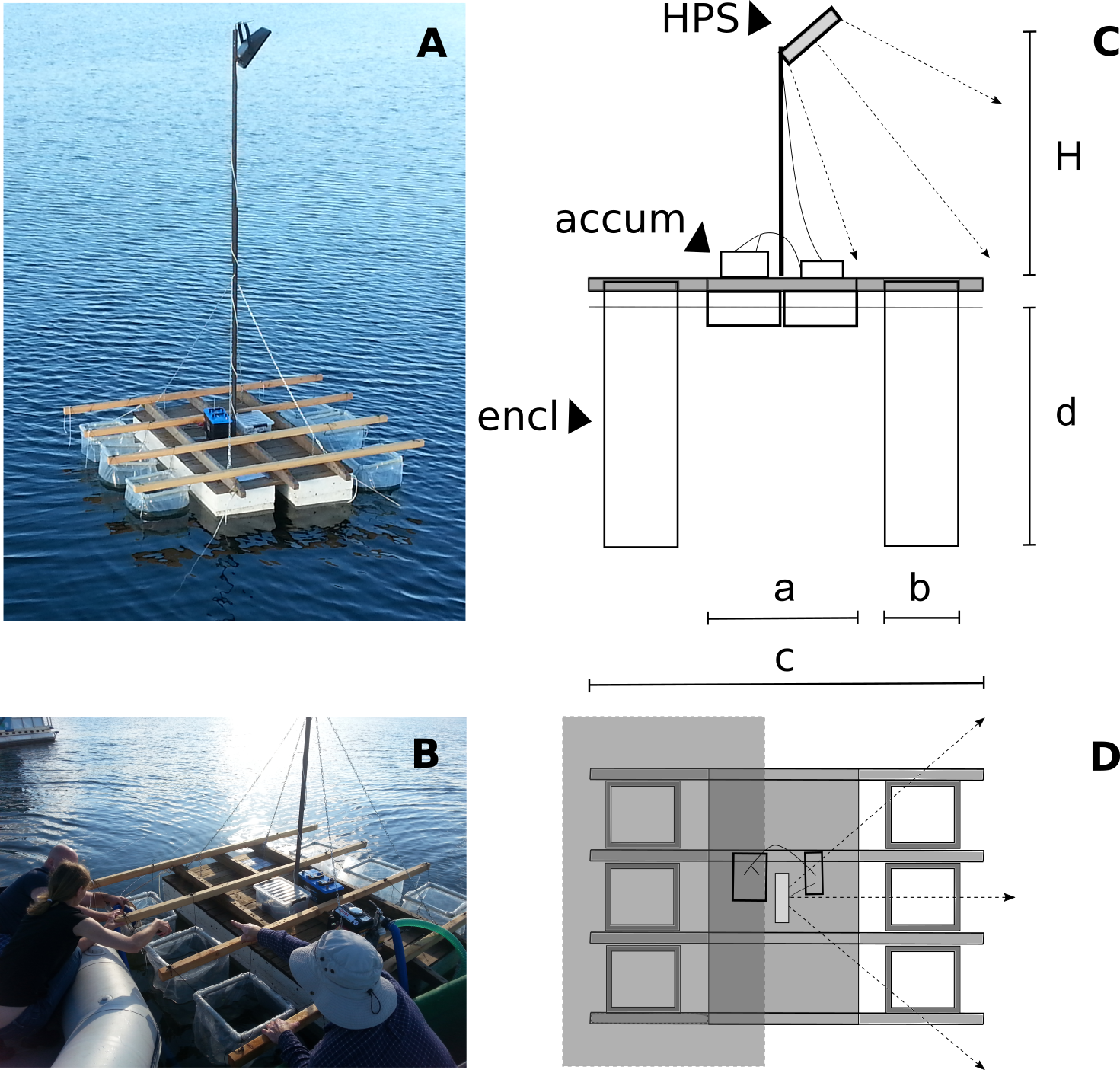


**Fig. 1.** The setup consisting of six enclosures attached to an anchored wooden platform with a spot artificial light source (HPS lamp) illuminating one side of the platform: (A) anchored in the experimental bay; (B) filling of enclosures with lake water; (C) schematic view from the side with measurements and details: (a) 1 m; (b) 0.5 m; (d) 7 m; (H) 2 m; encl – enclosure; accum – accumulator with voltage transformer; HPS – high pressure sodium lamp; (D) schematic view from above.
