# Supplementary material for: The short-term effects of planktivorous fish foraging in the presence of artificial light at night on lake zooplankton
Source: J Plankton Res. 2022 Sep 7;44(6):942–6. doi: 10.1093/plankt/fbac046 (PMC9692195; doi:10.1093/plankt/fbac046)
Supplement: Appendix_2_fbac046 [file appendix_2_fbac046.docx]

**Appendix 2.** **The details of the** **statistical analysis**

To test the ALAN effect on the: (1) final vertical distribution of each of the zooplankton taxa, (2) number of consumed prey representing each taxonomic group, (3) relative density changes of each of the zooplankton taxa as density after/before experiment, and (4) relative body length of *B. thersites* as their body length after/before experiment, we used a similar statistical approach. Due to the small number of replications (enclosures) and heterogeneous variances of the measured variables’ traditional parametric statistics were not pertinent for data comparison between the two treatments (“ALAN” and “dark”). Instead, the bootstrap approach was used to simulate the distributions of the analysed variables with the use of the collected data. The data were bootstrapped using the *“boot”* package, with 2000 samples per question and R statistical package (version 4.1.2).

The bootstrap was conducted with the strata specified, so that the number of observations coming from a given enclosure (and depth in case of the final vertical distribution analysis) was constant in each draw, yet was different for different parameters tested, e.g., 2 values in case of density or depth of animals from each enclosure or 14-16 values for fish gut content analysis in each enclosure. The 95% bias-corrected confidence intervals were calculated for the differences between the “ALAN” and “dark” enclosures.

Several analyses were performed on relative values considering initial values at the beginning of the experiment in each enclosure (e.g. taxon densities, body size of *B. thersites*). The ratio of final to initial value or mean was calculated for each enclosure prior to the bootstrap procedure. The residence depth of analysed taxons was compared between treatments upon weighted means calculated for each enclosure with densities in separate vertical strata used as a weight.

**Appendix 2, Table 1.** Bootstrap distributions of the differences in depth selected between the “ALAN” and “dark” treatments of each of the six taxa separately, the sum of four cladoceran species (*B. thersites, D.* *cucullata*, *L. kindtii*, *D. brachyurum*) and the sum of all six taxa. Statistically significant differences (according to 95% CI) are marked in bold.

|  | Original  difference | Mean bootstrap  difference | Low  95% CI limit | High  95% CI limit |
| --- | --- | --- | --- | --- |
| ***Cyclopoida*** | **-0.80** | **-0.80** | **-1.36** | **-0.36** |
| *Calanoida* | -0.04 | -0.05 | -0.41 | 0.30 |
| ***D. cucullata*** | **0.65** | **0.63** | **0.12** | **1.13** |
| ***B. thersites*** | **-0.86** | **-0.86** | **-1.18** | **-0.56** |
| *L. kindtii* | 0.33 | 0.20 | -2.55 | 3.15 |
| ***D. brachyurum*** | **-1.38** | **-1.36** | **-1.88** | **-0.84** |
| Sum of 4 cladoceran taxa | -0.14 | -0.15 | -0.50 | 0.15 |
| **Sum of all 6 taxa** | **-0.47** | **-0.46** | **-0.83** | **-0.13** |

**Appendix 2, Table 2.** Bootstrap distributions of the differences in the gut content of the fish from the “ALAN” and “dark” treatments of each of the six taxa separately, the sum of four cladoceran species (*B. thersites, D.* *cucullata*, *L. kindtii*, *D. brachyurum*) and the sum of all six taxa. Statistically significant differences (according to 95% CI) are marked in bold.

|  | Original  difference | Mean bootstrap  difference | Low  95% CI limit | High  95% CI limit |
| --- | --- | --- | --- | --- |
| *Cyclopoida* | -5.34 | -5.28 | -41.89 | 10.94 |
| *Calanoida* | -1.79 | -1.82 | -5.58 | 0.67 |
| ***D. cucullata*** | **119.87** | **118.86** | **55.94** | **207.51** |
| ***B. thersites*** | **265.63** | **263.73** | **151.42** | **458.14** |
| ***L. kindtii*** | **7.51** | **7.43** | **2.91** | **16.42** |
| ***D. brachyurum*** | **0.59** | **0.58** | **0.07** | **2.04** |
| **Sum of 4 cladoceran taxa** | **392.30** | **389.29** | **221.49** | **662.17** |
| **Sum of all 6 taxa** | **387.97** | **385.04** | **203.50** | **671.51** |

**Appendix 2, Table 3.** Bootstrap distributions of the differences in density between the “ALAN” and “dark” treatments of each of the six taxa separately, the sum of four cladoceran species (*B. thersites, D.* *cucullata*, *L. kindtii*, *D. brachyurum*) and the sum of all six taxa. Statistically significant differences (according to 95% CI) are marked in bold.

|  | Original  difference | Mean bootstrap  difference | Low  95% CI limit | High  95% CI limit |
| --- | --- | --- | --- | --- |
| *Cyclopoida* | -0.15 | -0.15 | -0.68 | 0.24 |
| ***Calanoida*** | **-0.50** | **-0.50** | **-1.27** | **-0.05** |
| *D. cucullata* | -0.21 | -0.21 | -0.55 | 0.04 |
| *B. thersites* | -0.21 | -0.21 | -0.76 | 0.19 |
| *L. kindtii* | -0.24 | -0.24 | -0.72 | 0.27 |
| ***D. brachyurum*** | **-0.87** | **-0.87** | **-2.11** | **-0.19** |
| Sum of 4 cladoceran taxa | -0.22 | -0.22 | -0.67 | 0.06 |
| Sum of all 6 taxa | -0.19 | -0.20 | -0.72 | 0.11 |
